# Hypoxic Stress-Dependent Regulation of Na,K-ATPase in Ischemic Heart Disease

**DOI:** 10.3390/ijms24097855

**Published:** 2023-04-26

**Authors:** Emel Baloglu

**Affiliations:** Department of Medical Pharmacology, School of Medicine, Acibadem Mehmet Ali Aydinlar University, 34752 Istanbul, Turkey; emel.baloglu@acibadem.edu.tr; Tel.: +90-216-500-4048

**Keywords:** Na,K-ATPase, ion transporter, hypoxia, ischemic heart, heart failure, HIF, cardiotonic steroids, cellular stress

## Abstract

In cardiomyocytes, regular activity of the Na,K-ATPase (NKA) and its Na/K pump activity is essential for maintaining ion gradients, excitability, propagation of action potentials, electro-mechanical coupling, trans-membrane Na^+^ and Ca^2+^ gradients and, thus, contractility. The activity of NKA is impaired in ischemic heart disease and heart failure, which has been attributed to decreased expression of the NKA subunits. Decreased NKA activity leads to intracellular Na^+^ and Ca^2+^ overload, diastolic dysfunction and arrhythmias. One signal likely related to these events is hypoxia, where hypoxia-inducible factors (HIF) play a critical role in the adaptation of cells to low oxygen tension. HIF activity increases in ischemic heart, hypertension, heart failure and cardiac fibrosis; thus, it might contribute to the impaired function of NKA. This review will mainly focus on the regulation of NKA in ischemic heart disease in the context of stressed myocardium and the hypoxia–HIF axis and argue on possible consequences of treatment.

## 1. Introduction

The Na,K-ATPase (NKA) enzyme or sodium potassium pump, a member of the P-type ATPase family, was first described by J.C. Skou in 1957, who was the first to link the well-known Na^+^-dependent ATP hydrolysis and energy-dependent Na^+^/K^+^ exchange to the one enzyme [1]. Since its discovery it has been an attractive topic for understanding its contribution to physiological functions and pathological conditions as it is ubiquitously expressed in all mammalian cells and tissues.

The well-defined function of NKA is that it transports 3Na^+^ ions out of the cell and 2K^+^ ions into the cell with the energy obtained from hydrolysis of ATP. It thus contributes to the maintenance of the cellular homeostasis of Na^+^, K^+^ and, indirectly, of Ca^2+^ by modulating the activity of the Na^+^/Ca^2+^ exchanger (NCX) [2]. Thereby, it also determines the activities of other secondary active transporters such as Na^+^/H^+^ and Na^+^-HCO_3_^−^ exchangers, whose activities depend on Na^+^-gradients, and affects intracellular pH as well as glucose and amino acid transport into the cells [3]. In the heart muscle it plays a critical role in the generation of action potentials, electrical activity, maintenance of Na^+^ and Ca^2+^ ion gradients, regulation of intracellular volume, and cell survival. All these functions make the NKA an important signaling molecule for proper function of cardiomyocytes and an important target for the treatment of heart failure.

Recent studies identified new roles of cardiotonic glycosides, the inhibitors of the NKA defined first by Schatzmann in 1953 [4], which have been used as positive inotropic agents in the treatment of congestive heart failure for more than two hundred years. Our current understanding is that NKA is not simply an ion-transporter, but it is also involved in regulating collagen synthesis in cardiac fibroblasts [5], modulating intracellular Ca^2+^, signaling via c-Src mechanisms in vascular smooth muscle cells [6], and increasing gene transcription by protein kinase C-dependent mechanisms in cardiomyocytes [7]. These studies imply yet unidentified roles of the NKA that need to be uncovered.

Various studies reported decreased activity of the NKA in ischemic heart disease models and attributed this blunted activity to decreased expression or rearrangements of the subunits, though contradictory findings exist [8,9,10,11,12]. There are also reports about redox-dependent post-translational regulation of the enzyme [13], and cellular redox changes that affect its activity [14].

A common finding of the ischemic heart is hypoxia of the myocardium. Under low oxygen tension, hypoxia inducible factors (HIF) control adaptation of the cells by switching on and off different signaling mechanisms. HIF activity increases in the ischemic heart, systemic and pulmonary hypertension, heart failure and cardiac fibrosis. Yet, no study has sought to address and link HIF in regulating NKA in a “stressed hypoxic myocardium”.

A more detailed understanding on the regulation of NKA and consequences in the ischemic heart is required to reveal the changes that occur in the hypoxic myocardium, which may also expand different perspectives and treatment strategies against heart failure and cardiac arrhythmias. The aim of this review is to summarize the current knowledge on the regulation and activity of NKA with particular emphasis on the ischemic heart and question any likely involvement of HIF in the content of hypoxia/ischemia and further treatment strategies.

## 2. Ischemic Heart Disease

Cardiovascular system diseases (CVDs) are the leading cause of mortality and morbidity in the world [15]. CVDs consist of multifactorial pathophysiological changes in the heart and circulation, represented by heterogeneous clinical presentation. About 85% of CVD-related deaths are the result of cardiac ischemia and stroke.

Ischemia of the heart is the inadequate perfusion of myocardial tissue due to decreased oxygen and nutrient supply and inefficient removal of metabolic end products. The most common factor is occlusion of coronary arteries with a thrombus. Short-term ischemic attacks can be tolerated well, whereas long-term ischemia can cause persistent and progressive effects due to the remodeling of blood vessels and myocardium by increased activity of the renin-angiotensin-aldosterone and sympathetic systems, contributing to the development of cardiac fibrosis and heart failure.

Myocardial oxygen supply is also impaired in anemia, pregnancy, and in diseases such as myocardial infarction, pulmonary and systemic hypertension, and hypertrophic heart failure due to increased cardiac work and myocardial oxygen demand that exceeds the oxygen delivery.

Treatment modalities against ischemic heart disease vary, depending on the type of underlying disease, which are represented by a wide range of symptoms. Current pharmacological treatments directed to the altered pathological signalling changes include vasodilators, positive inotropic agents, diuretics, angiotensin converting enzyme inhibitors, angiotensin receptor blockers, beta adrenergic and mineralocorticoid receptor antagonists, and myofilament calcium sensitizers. These agents are still used with limited benefits. Hence, there is still a need to develop new treatment approaches [16]. Therefore, unravelling the pathological changes in the ischemic heart in depth will shed light on better future drug development and treatment strategies.

## 3. Structural Properties of Na,K-ATPase (NKA)

**Figure 1:**
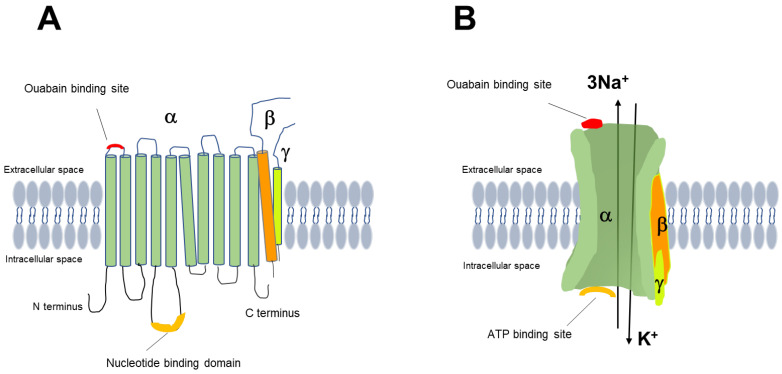
Structure (**A**) and function (**B**) of NKA.

NKA is a hetero-oligomeric protein and consists of three different subunits: the catalytic α subunit, a regulatory β subunit and a small γ subunit, which is a member of the FXYD proteins involved in regulating α-NKA (structure and function of NKA has been shown in Figure 1).

### 3.1. α-NKA

The α subunit is a ten transmembrane helices protein and has binding sites for Na^+^, K^+^, Mg^2+^, ATP and cardiac glycosides [17,18]. There are four known isoforms of the NKA-α subunit (α1, α2, α3 and α4), which are expressed at different levels in various tissues and species [19]. α1-NKA is expressed in all cells, and the α2 and α3 isoforms are expressed mostly in the heart, skeletal muscle, and neurons. The α4 isoform is found in the testes, where it is required for sperm fertility [20,21]. Human ventricular myocardial cells express α1, α2 and α3 subunits whereas mouse and rat myocardium express only α1 and α2 subunits; with the α1 subunit being the ubiquitous form [22]. In heart ventricular myocytes α1-NKA is densely and diffusely located in the plasma membrane, whereas α2 and α3 subunits are mostly found in T-tubules [23,24]. To date, the structure of α1-NKA has been identified and described as having three cytoplasmic domains: the nucleotide binding (N), the phosphorylation (P) and the actuator (A) domains. The N domain functions as kinase, the P domain is the substrate binding domain and the A domain has the phosphatase activity that hydrolyzes ATP [25].

### 3.2. β-NKA

The β-NKA is a single transmembrane protein and has three isoforms (β1, β2 and β3) expressed with different abundancy depending on the tissue [25]. The β isoform has glycosylated and non-glycosylated forms. In the heart three isoforms are expressed. Recent studies identified their crucial roles in the structural maturation of NKA and modulation of the activity. The most studied, the β1 isoform, which is a small protein with molecular chaperone functions, controls the correct orientation and membrane insertion of α1-NKA [19], and it is therefore required for the functionality of NKA. The roles of other subunits have not been identified yet. It may be likely that they have similar functions depending on the tissue. The functional determinant of the pump is attributed to αβFXYD combination with a 1:1:1 stoichiometry. According to Clifford and Kaplan, multiple isoforms of β subunits may interact with the α1 subunit in MDCK cells [26]. This different subunit organization of NKA isoforms in various tissues allows optimized NKA activity [20,25] and different sensitivity to substrates, cardiac glycosides and various stressors [27].

### 3.3. FXYD Proteins

The mammalian FXYD proteins (FXYD1-7) are a family of seven small membrane-spanning proteins which are abundantly expressed and associate with and modulate the function of NKA [28,29]. In the heart FXYD1, also known as phospholemman (PLM), is expressed. Unphosphorylated PLM inhibits NKA activity by interacting with α and β subunits whereas phosphorylated PLM by protein kinase A (PKA) increases NKA activity in ventricular myocytes [29,30].

The operating cycle of NKA is mediated by two conformations, E1 and E2, which denote the high affinity for Na^+^ and K^+^, respectively. The pump is at the E1 state when it has high affinity for Na^+^ and ATP. After the hydrolysis of ATP, the P domain is phosphorylated and the 3Na^+^ ions are occluded in the binding region. The transition from phosphorylated E1 (E1P) to E2P occurs by the release of ADP, opens the outer gate and releases 3 Na^+^ ions to the extracellular side. This conformation increases the binding affinity of K^+^ from the extracellular side, occludes 2K^+^, and then the P domain is dephosphorylated (E2). During the ion pumping cycle and cardiac glycoside binding, the relative positions of these domains change [31]. Accurate persistence of each cycle depends on the proper levels of Na^+^, K^+^, ATP and expression and organization of the pump subunits.

## 4. Role of Na,K-ATPase (NKA) in Cardiac Excitation–Contraction Coupling

Cardiac impulses originate in the sinus node, and propagation in the heart depends on the magnitude of the depolarizing current. Once impulses leave the sinus node, they propagate rapidly throughout the atria, flowed by the AV node, spread from the His-Purkinje system on the endocardium of the ventricles throughout the rest of the ventricles, stimulating coordinated ventricular contractions. 

The most important ion involved in regulating cardiac contraction (systole) and relaxation (diastole) is intracellular Ca^2+^. In the healthy heart, voltage-dependent Na channels open after a depolarization wave generated from sinoatrial node arrives to the cardiomyocytes and leads to increased intracellular Na^+^ levels. Depolarization enhances the opening probability of L-type voltage-dependent Ca channels which are densely located at T-tubules. A small amount of Ca^2+^ enters via L-type voltage-dependent Ca channels, binds to ryanodine receptors (RyR) located at the endoplasmic reticulum (ER), and triggers the release of more Ca^2+^ to cytosol, resulting in the so-called Ca^2+^-dependent Ca^2+^ release. Increased intracellular Ca^2+^ binds to the Ca-binding subunit of the thin filament protein troponin C on the myofilament, activates interactions between actin and myosin that result in sarcomere shortening, triggering contraction [18]. In addition, Ca^2+^ binds to the ancillary calcium-binding protein calmodulin, which activates myosin light chain kinase (MLCK), and in turn phosphorylates the regulatory light chain of myosin, causing contraction.

In diastole, Ca^2+^ is released from the contractile proteins, binds to SERCA, a Ca-ATPase located at the ER and is taken up for storage, thus rapidly decreasing the intracellular Ca^2+^ concentration. Other mechanisms removing excess amounts of intracellular Ca^2+^ are NCX, which uses the electrochemical gradient of intracellular Na^+^, and the ATP-dependent Ca^2+^-ATPase, located in the plasma membrane [32]. Therefore, intracellular Na^+^ levels must be kept within certain limits to maintain intracellular Ca^2+^ homeostasis at physiological levels [33].

Depending on the Na^+^ gradient generated by the NKA, Na^+^ enters cardiomyocytes via voltage-gated Na channels, NCX, Na^+^/H^+^ exchanger (NHE), Na^+^/Mg^2+^ exchanger Na^+^/HCO_3_^−^ and Na^+^/K^+^/2Cl^−^ cotransporters. The only mechanism that extrudes Na^+^ from the cells is the NKA pump [34]. Voltage-gated Na channels together with exchangers and (co)transporters must work compatibly and evenly to determine the balance of intracellular Na^+^ which also indirectly affects the intracellular Ca^2+^ level. Therefore, any change that may directly affect the proper activity of NKA or indirect changes may consequently impact optimal heart function. 

## 5. Na,K-ATPase (NKA) in Ischemic Heart Disease

Understanding the underlying mechanisms of NKA regulation in ischemic heart diseases has been an important issue owing to its essential functions for myocytes. Many studies reported decreased activity of NKA in tissues from the ischemic heart and in animal models of ischemic heart disease [35]. In parallel to decreased pump activity, intracellular Na^+^ levels were found elevated due to profound activity of voltage sensitive Na channels and NHE, which dominate over NKA activity [36]. In animal models with heart failure and in patient tissues, elevated Na^+^ increases the activity of NHE and NCX, deteriorates diastolic function, causes arrhythmia, compromises cell metabolism and induces oxidative stress [10,18,37].

### 5.1. Studies in Human Tissue Samples with Ischemic Heart Disease

Various reports proposed and associated the impaired activity of NKA with decreased expressions of α1, α3 and β1 subunits [8,9]. Ishino et al. suggested acute cardiac decompensation leading to progressive pump failure as the main cause of death in patients with congestive heart failure. They measured cellular ATP content and total NKA expression in right ventricle biopsies along with left ventricular ejection fraction (LVEF) from 23 patients with irreversible cardiogenic shock compared with 20 patients who had compensated heart failure. In patients with irreversible cardiogenic shock, cellular ATP level and NKA expression was lower than the compensated heart failure group, and deteriorated LVEF was significantly correlated with ATP and NKA. This study further suggested that decreased ATP and NKA could partially contribute to the development of spontaneous deterioration of a chronically overloaded heart. However, relative changes of subunits’ expression and NKA activity have not been measured [38]. Norgaard et al. measured [3H]-ouabain binding in left ventricle biopsy samples from 19 dilated cardiomyopathy patients with impaired LVEF and reported decreased NKA concentration compared to those with normal left ventricular function. The decreased NKA concentration was correlated with systolic dysfunction. Furthermore, in 16 of patients receiving digoxin, NKA concentration and ejection fraction showed a significant correlation, indicating that the decrease in NKA concentration in patients with impaired LV function was not due to accumulation of digoxin in the myocardium. However, this study did not focus on NKA expression and cellular ATP content [39]. It is known that cellular ATP levels decrease during ischemia, but this does not seem to be the main factor limiting the NKA activity, since NKA activity decreases before the decline in cellular ATP and the levels are high enough to drive NKA [40,41].

Studies measuring NKA activity in necropsy materials from heart failure patients found about a 40% decrease [10]. However, results on mRNA and protein level are inconsistent [10,11,12]. Schwinger et al. showed decreased NKA activity by 43% and [3H]-ouabain binding by approximately 40% in the left ventricles of heart failure patients. In this study they found decreased protein levels of α1-, α3- and β1-NKA in the left ventricles of failing myocardium compared with non-failing myocardium. They did not find any change in α2-NKA protein level [9]. Similarly, Shamraj et al. reported decreased [3H]-ouabain binding but no alteration in isoform compositions [42]. In another study, Allen et al. reported no change in the mRNA expression of α1, α2 and α3 subunits and ouabain binding between normal hearts and end-stage heart failure patients [43]. In patients with systemic hypertension, Jager et al. showed no change in the mRNA expression of α1- but increased α2- and α3-NKA subunits. However, in this study they did not measure protein-level and activity of the NKA [44]. These findings suggest that NKA activity decreases in end-stage heart diseases, but mRNA and protein expression of the subunits vary and collectively seem not to explain the changes in activity. The observed inconsistencies in NKA subunit expression might be due to different locations where the biopsy samples were taken; the age, sex [45], and backgrounds of the patients; their disease type and state; and the used treatments. The latter may cause additional bias to the observed discrepancies since end-stage patients are required to receive positive inotropic support such as cardiac glycosides.

### 5.2. Studies in Animal Models of Ischemic Heart

Regarding the activity and expression of NKA, studies performed on different animal models of ischemic heart disease provided more interpretive details compared to findings from human tissue samples with ischemic heart disease. In tissue homogenates from heart failure models with different species and experimental approaches, NKA activity was found to be decreased by ~30% [10]. Semb et al. showed decreased capacity of NKA with no change in the mRNA and protein expressions of α1- and β1-NKA in a post-infarction model of hypertrophy and congestive heart failure in rats. In this study they found an isoform switch from α2- to α3-NKA due to increased protein expression of α3-NKA [8]. Bossuyt et al. reported decreased α1 and α2 isoforms in ventricular homogenates (by 24%) and isolated myocytes (by 30% and 17%, respectively) from rabbits with heart failure. In this study the α3 isoform was found to be increased (50%) in homogenates but decreased (52%) in myocytes. Despite these differences in subunit patterns, intracellular Na^+^ levels increased because of decreased NKA activity, which correlated with increased protein levels of phosphorylated phospholemman (PLM) fraction and its association with α subunits [11]. This study not only points at the importance of studying isolated myocytes rather than tissue homogenates but also interacting protein partners that regulate the NKA activity.

However, there are limitations about the NKA expression in these studies since the expression of the subunits in animal models and human necropsy materials mostly have been performed in frozen whole heart tissue homogenates, crude membrane fractions or whole cell lysates from isolated cells. These preparations do not reflect the pure membrane fractions where active and mature pump subunits are located, and they also contain cytoplasmic and lysosomal fractions. They give limited and general information about the total expression patterns of the subunit proteins. Therefore, the relative changes in the membrane, intracellular expression, and localizations of the subunits need to be investigated in more detail even including intracellular compartments.

As mentioned above, some studies reported an isoform switch from α2- to α3-NKA in cardiac hypertrophy and failure models. Of note, reported shifts of the subunits in the ischemic heart affects the activity of the NKA because in the rodent heart the α1 isoform is less sensitive to ouabain than the α2 isoform [46]. Since a high concentration of ouabain is required to inhibit the α1 isoform in rodents, the sensitive α2 isoform is also inhibited and the contribution of the α1 isoform to contractility can be masked [47,48,49]. To dissect the contribution of α2-NKA in pump function and ouabain sensitivity in transgenic mice expressing the ouabain-insensitive α2 isoform of NKA, Dostanic et al. reported that a non-toxic dose of ouabain increased cardiac contractility via α2-NKA in vivo. [50]. Moseley et al. used α1^+/–^ transgenic mice expressing 50% less protein abundance with an increased α2 subunit due to compensation and showed the reduced contractility to a high dose of ouabain and an increased contractility to a low dose [51]. However, these models have not addressed the effect of ouabain response in cardiac ischemia models and cellular localization of the NKA subunits.

It is reported that in T-tubules of cardiomyocytes α2-NKA assembles with β2-NKA and localizes in close proximity to NCX; it might thereby indirectly contribute to intracellular Ca^2+^ regulation [52]. The latest studies suggest that α1-NKA is the “housekeeping” subunit regulating global Na^+^ [53], whereas defined localizations of α2-NKA at T-tubules might specifically regulate Na^+^ locally in distinct functional domains shared with NCX [54]. Alternatively, Dostanic et al., using genetically engineered knock-in mice expressing ouabain-sensitive α1 and ouabain-resistant α2 isoforms of NKA, showed both α1 and α2 isoforms co-localized with NCX and responded to low and high doses of ouabain; further suggesting that both isoforms play similar roles in regulating cardiac contractility [55]. To determine whether maintaining lower intracellular Na^+^ would enhance Ca^2+^ clearance via NCX, Correll et al. used cardiac-specific transgenic mice overexpressing α1- or α2-NKA and subjected them to pressure overload hypertrophic stimulation. They found that mice overexpressing α2-NKA had decreased cardiac hypertrophy, both acute and long-term, after pressure overload, increased NKA activity, enhanced removal of Ca^2+^ from the cytosol via NCX1 without any change in intracellular Na^+^ level, decreased PLM expression and phosphorylation, and suggested protective effects [56]. In a recent study Cellini et al. used cardiomyocyte-specific overexpressing α2-NKA which developed myocardial infarction. They showed that mice overexpressing α2-NKA improved cardiac contractility, reduced cardiomyocyte hypertrophy and end-diastolic dimensions, and increased beta1 adrenergic receptor levels compared to wild type mice [57]. Studies using transgenic overexpression of the α2 isoform point to protective effects in various ischemic heart models. However, questions arise whether similar effects also occur in non-overexpression systems and in the conditions where isoform switch occurs to counterbalance the decreased α1 isoform.

## 6. Hypoxia and Hypoxia Inducible Factors (HIFs) as Hallmarks of Ischemic Heart Disease

### 6.1. Regulation and Signaling of Hypoxia Inducible Factors (HIFs)

Molecular oxygen is vital for survival. The requirement of physiological oxygen level of each cell is different. Oxygen is transported by binding to hemoglobin and is delivered to tissues via systemic circulation. Many diseases of the lung and cardiovascular systems are associated with tissue hypoxia. Under hypoxic conditions, some physiological processes are activated such as the sympathetic system to increase blood flow to the ischemic region; thus oxygen transport and delivery [58].

Hypoxia inducible factors (HIFs) are key proteins that control the adjustment of cells to low oxygen levels by modulating the transcription of some genes. HIFs are expressed in all eukaryotic species and contribute to the embryonic developmental stages, physiological functions, and pathological processes of living organisms [59]. All types of cells sense hypoxia, but their response may differ. When oxygen supply is decreased, cells typically switch from aerobic to anaerobic metabolism to warrant ATP production. Other oxygen-dependent signals initiate systemic responses such as increased respiratory rate, angiogenesis, and erythrocyte production by secreting metabolites and hormones to increase oxygen delivery to the body [60].

In structure, HIF is a heterodimer and consists of an oxygen-regulated α subunit (HIF-1α, HIF-2α, HIF-3α) and a constitutively expressed β subunit [61]. HIF-α is barely detectable under normoxic conditions [62] because it has a very short half-life, and its level increases depending on the duration and severity of hypoxia and other cellular stress signals. 

HIF-α levels depend on the activity of the enzyme HIF-prolyl hydroxylase (PHD). In the presence of oxygen, PHD hydroxylates two prolyl residues in the HIF-α. This permits the binding of the Von Hippel-Lindau tumor suppressor protein (pVHL), an E3 ubiquitin ligase, leading to degradation by the 26S proteasome system [63]. Under low oxygen conditions, a hydroxylation reaction cannot occur and HIF-α becomes stabilized. It forms a dimer with HIF-β, a subunit that is constitutively expressed. The dimer translocates to the nucleus and binds to the so-called hypoxic response element (HRE), a specific base-sequence in the promoter region of oxygen-controlled genes, to initiate their expression [64]. Although HIF-1α and HIF-2α increase the transcription and protein synthesis of some genes, this effect may vary from cell to cell. Some of these genes are vascular endothelial growth factor (VEGF; involved in angiogenesis), angiopoietin 1 and angiopoietin 2 (ANGPT1, ANGPT2; regulator of angiogenesis and vascular permeability) [65], lactate dehydrogenase (LDK1; converts pyruvate to lactate) [66], pyruvate dehydrogenase kinase (PDK1, PDK3; inactivates pyruvate dehydrogenase and shunts pyruvate away from the mitochondria) [67], glucose transporters (GLUT; increases glucose uptake), peroxisome proliferator activated receptor (PPAR-γ; increases lipid synthesis) [68], Bcl-2/adenovirus E1B 19-kDa interacting protein 3 (BNIP3) and Bcl-2/adenovirus E1B 19-kDa interacting protein 3-like protein (BNIP3L; trigger mitochondria selective autophagy) [59].

### 6.2. Hypoxia Inducible Factors (HIFs) in the Ischemic Heart

HIFs are not only active in adult life but also during embryonic development, where all organs, and thus also the heart, develop in a low-oxygen environment [69]. In the early embryonic period, cardiac myocyte precursors are dependent on glycolytic pathways and pyruvate oxidation as an energy source. As the heart grows and develops towards an adult heart, the energy demand increases. This is achieved by the development of mitochondria and oxidative capacity transition from glycolytic pathways to oxidative phosphorylation for a high energy source [70].

In an anaerobic environment, adult heart cells cannot maintain their viability for. The first adaptation mechanism of the heart to ischemia is to maintain the intracellular ATP homeostasis. This is achieved by transferring the energy obtained from the beta oxidation of fatty acids produced in the “adult type” heart to glycolytic pathways. During this transition period, expression and activities of enzymes involved in beta oxidation decrease, those functioning in the glycolytic pathway increase because of a mismatch between myocardial oxygen demand and supply [71,72]. Similar changes also occur in cardiac hypertrophy and heart failure induced by pressure and volume overload [73,74,75,76]. In human and mouse models of cardiac hypertrophy, glycolytic metabolism is turned on due to increased HIF-1α [68]. However, this vital adaptive mechanism has a bidirectional effect on myocyte survival under stress conditions. For example, long-term elevation of HIF levels may cause apoptosis and cardiac decompensation, whereas short-term activation of HIF-1α has protective effects [59,68], indicating the fine-tuned temporal activity of HIFs is critical in regulating adaptive mechanisms during cardiac ischemia.

**Figure 2:**
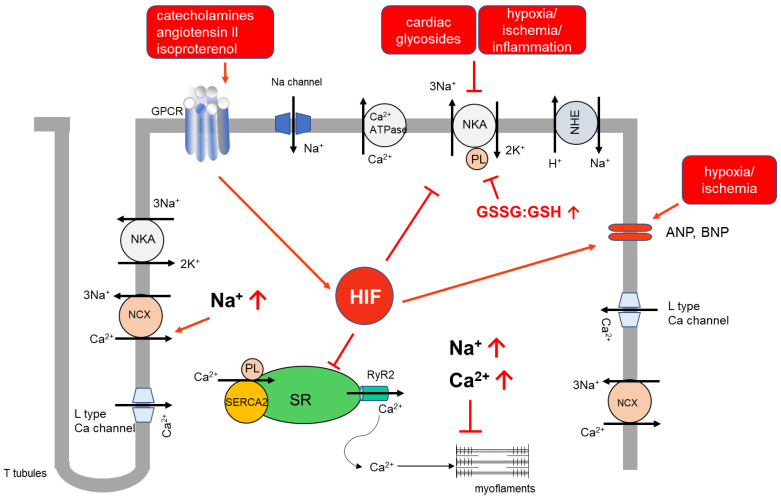
Modulation of NKA activity in an ischemic heart. Catecholamines, angiotensin II and isoproterenol stimulate relevant receptors, increase HIF, and their levels gradually increase during the progression of heart failure. Increased HIF might be involved in regulating NKA in the ischemic heart. Hypoxia, by changing the redox status of cardiomyocytes, causes posttranslational modifications of the NKA subunits, and by decreasing membrane expression of the subunits inhibits NKA. Inflammatory cytokines via PI3K/Rac1/NADPH oxidase cascade inhibits NKA. Furthermore, cardiotonic steroids inhibit NKA, leading to elevated intracellular Na^+^ and Ca^2+^ levels, which further deteriorate cardiac contraction and function.

**Figure 3:**
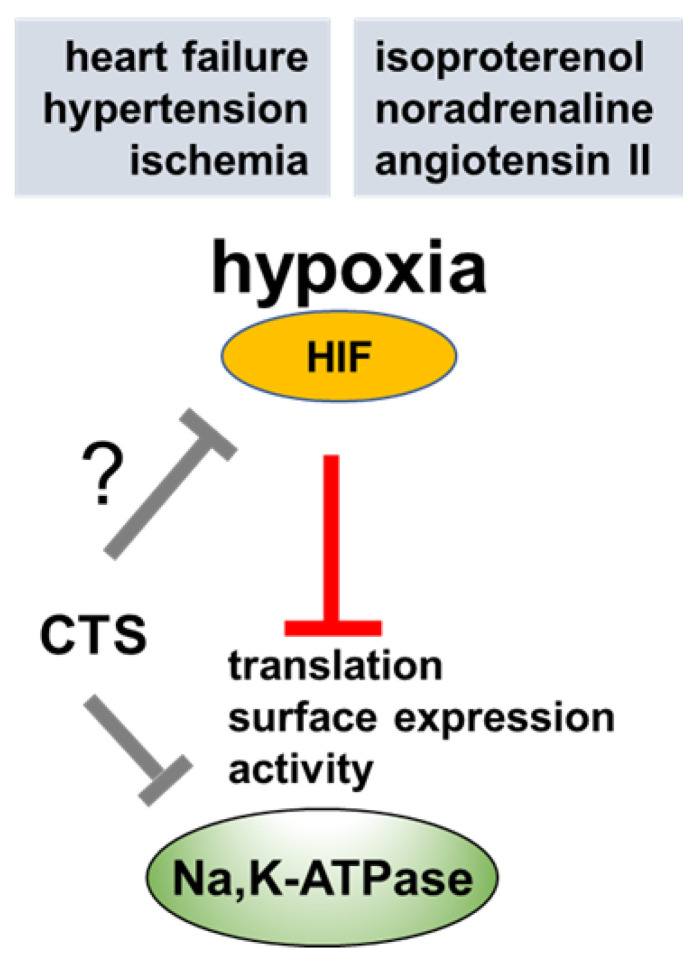
Possible regulation of NKA by HIF in the hypoxic myocardium. HIF: hypoxia inducible factor; CTS: cardiotonic steroids.

Increased HIF-α expression and activity has been reported in many CVD (recently reviewed [77]). In human tissues and in mouse models of cardiac hypertrophy induced by aortic constriction, HIF-1α levels increased [68]. Interestingly, knock-down of HIF-1α prevented cardiomyocyte apoptosis and contractile dysfunction. Similarly, increased HIF-α expression also occurred during cardiac remodelling [78]. In humans and animals with pulmonary hypertension, HIF-1α and HIF-2α levels increased in vascular smooth muscle cells and lung vascular endothelial cells, respectively [79]. Isoproterenol-induced myocardial arrhythmia increased HIF-1α expression in atrium [80]. In animal models of myocardial infarction by coronary ligation or systemic hypoxia, the accumulation of HIF-1α and HIF-2α persisted for several weeks [81]. Lee et al. reported the increased expression of HIF-1α and VEGF in myocardial ventricular-biopsy specimens from patients undergoing coronary bypass surgery [82]. Cowburn et al. showed the correlation between HIF-α expression in human skin and its association to idiopathic hypertension by NO-dependent mechanisms [83]. In many in vitro and in vivo ischemic heart, cardiac hypertrophy and fibrosis models, the commonly used agents noradrenaline and angiotensin II increased cardiac work and oxygen demand (Figure 2 and Figure 3). These treatments elevated HIF-1α levels, produced HIF-dependent metabolic changes and caused pathological remodelling [68,80,84]. These agents also have been widely used in studies focusing on the regulation of NKA activity in the ischemic heart. Given that the increased levels of HIFs are hallmarks of hypoxia and of cardiovascular disease, it is likely that HIFs might be also involved in regulating the activity and expression of NKA in the ischemic heart. This aspect needs further experimentation.

## 7. HIF-1α in Regulating NKA in the Ischemic Heart

Hypoxia inhibits the transcription and translation of some proteins to adapt and allow cells to consume less ATP for maintenance of their viability and survival. HIF-dependent modulation of activity and expression of membrane receptors and ion channels, including NKA, has been shown in different cells and tissues in hypoxia. Xie et al. reported that von Hippel-Lindau tumor suppressor protein (pVHL)-E3 ligase complex, which regulate HIF-α expression, interacted and ubiquitylated beta2 adrenergic receptor (β2AR), and decreased the membrane expression in hypoxic cells [85]. Another study showed PHD2-dependent internalization of β2AR into cytosol in normoxic cells [86]. Recchia et al. reported HIF-1α-dependent increased expression and activity of G protein-coupled receptor 30 (GPR30) in breast cancer cells and in HL-1 cardiomyocytes [87]. Magnani et al. showed that stabilization of NKA during severe hypoxia is an HIF-dependent process and involved protein kinase C zeta (PKC ζ) degradation in alveolar epithelial cells [88]. Our group recently showed that HIF-2α is involved in preventing internalization and degradation of surface expression of epithelial Na channels (ENaC) in primary alveolar epithelial cells exposed to in vitro hypoxia, and it improved the amiloride-sensitive alveolar fluid reabsorption, which was decreased in in vivo hypoxia [89]. Ochoa et al. recently reviewed the regulation of the calcium and voltage-activated potassium channel (BK) by hypoxia, HIF-1α and its relation with CVD [90]. Most of these studies have been performed in vitro; thus, whether similar regulations by HIFs also occur in vivo still needs to be identified.

Other important signaling molecules modulating NKA activity are atrial natriuretic peptide (ANP) and brain derived natriuretic peptide (BNP) which have vasodilatory, natriuretic, antihypertrophic, antifibrotic and other cardiometabolic effects [91]. ANP and BNP are produced and released from atrium and ventricles in response to mechanical, hemodynamic, humoral, ischemic, and inflammatory inputs and their blood levels increase in ischemic heart disease [92]. Today, monitoring blood levels of BNP together with cardiac troponins T/I became standard clinical biomarkers for the diagnosis, prognosis and evaluation of cardiovascular risk of patients with cardiac diseases, including ischemic heart disease and heart failure [93,94,95].

Studies revealed the mechanisms of ANP and BNP production and secretion from the ischemic heart. In regional ischemia of a rat heart model by coronary ligation, Chun et al. showed induction of ANP transcription and elevated HIF-1α protein compared to a non-ischemic region. This study also identified an HIF-1α binding site on the ANP promoter in rat ventricular H9c2 cells and primary neonatal cardiomyocytes [96]. Weideman et al. reported direct induction of BNP mRNA and its HIF-dependency in in vivo and in vitro hypoxia-exposed adult cardiomyocytes [97]. These studies showed that both ANP and BNP are produced and released from the ischemic heart in an HIF-1α-dependent manner. 

The Kidney is an important organ where ANP mediates diuretic and natriuretic effects. To dissect the signaling mechanisms behind this, Scavone et al. showed that in the kidney ANP inhibited NKA activity by cGMP mediated protein kinase G (PKG)-dependent phosphorylation of the protein [98]. The effects of ANP on NKA in the heart have been shown by patch clamp experiments using isolated cardiomyocytes and found increased NKA activity by NO-inducing soluble guanylate cyclase (sGC) and PKG with specific activators and inhibitors [99,100]. This data indicates that different effects of ANP may be attributable to tissue type regulation of NKA. However, there is no information about the effects of ANP on NKA in the ischemic heart, which needs to be addressed in future studies (Figure 2).

Studies focusing on the role of HIFs in controlling NKA activity in the ischemic heart are scarce. So far, some studies have focused on the role of HIF on Ca^2+^ signaling in hypoxia. In a rat model of chronic renal failure by partial nephrectomy, which caused cardiac hypertrophy, Kennedy et al. reported decreased activity of NKA along with α1- and α2-NKA mRNA and protein level SERCA2 expression and activity. In this study they also found prolonged intracellular Ca^2+^ recovery, contractile dysfunction and impaired relaxation in isolated cardiomyocytes [101]. Ronkainen et al., in hypoxic embryonic mouse cardiomyocytes, showed decreased SERCA2a expression and activity, impaired cardiac contractility and arrhythmia due to elevated intracellular Ca^2+^ levels. Overexpression of HIF-1α in normoxic cells and chemical activation of HIF-1α mimicked the findings induced by hypoxia and identified HRE binding sites in the SERCA2 promoter region [102,103]. 

CaMK2 is a key transducer of calcium signals in the heart both in physiological and pathological settings. In a myocardial infarct model, CaMK2γ expression decreased due to elevated HIF-1α levels, pointing to its important role in calcium signaling and transcriptional response to hypoxia [104].

In an in vitro ischemic heart model using the H9c2 rat ventricular cell line, we observed that 24 h of hypoxia (1% O_2_) decreased mRNA expressions of α1-NKA and β1-NKA by 30% and 20%, respectively (unpublished results [105]). Silencing HIF-1α in hypoxic cells totally prevented this effect, implying the possibility of HIF-1α in regulating NKA expression in the ischemic heart. These findings indicate that more comprehensive studies on the regulation of NKA are required to uncover the signaling in in vivo and in vitro models of hypoxic cardiomyocytes. 

## 8. Redox and Inflammation-Dependent Regulation of NKA

HIF-α is not only regulated by continuous hypoxia by classical signaling pathways, but also by intermittent hypoxia [59]. In this latter case the signal increasing HIF-α is not hypoxia but ROS generated upon reoxygenation. It is known that the activity of NKA is affected by hypoxia, hyperoxia, oxidative stress and by the cellular redox status. ROS sensitivity has been shown for α1- and β1-NKA, which both contain several cysteine residues in structure. The β2 and β3 isoforms do not contain cysteine residues and therefore are not subject to oxidative thiol modifications [106].

### 8.1. Glutathionylation of NKA Subunits by Cellular Redox Status

Hypoxia increased oxidized glutathione (GSSG) [107] causes glutathione depletion and diminishes antioxidant defense mechanisms. This increases the modification of thiols in cysteine residue within the ATP binding pocket of the catalytic α subunit of NKA, interfering with ATP binding to the nucleotide binding domain [107]. In α-NKA, reversible S-glutathionylation protects the protein from irreversible oxidative damage to preserve its function [107,108]. S-glutathionylation of α-NKA is maximal in the E1 but less so in the E2 conformation, and not all cysteine residues of α-NKA are available for this post-translational modification [109]. When the thiol groups in α1-NKA are irreversibly oxidized, NKA undergoes degradation [49] and the activity is associated with the decreased glutathione level of the cells.

Some studies reported that oxidation of the thiol groups of NKA did not cause inhibition of NKA because even high concentrations of hydrogen peroxide did not affect the NKA activity although the protein is fully oxidized [110]. However, during prolonged hypoxia, oxidative enzyme capacities, NO, and ROS levels decrease, along with increased GSSG. These changes are accompanied by decreased S-nitrosylation and increased S-glutathionylation of the α1-NKA [107]. Therefore, besides oxidation of the thiol groups during hypoxic stress, other changes such as GSSG/GSH ratio and cellular redox status may be responsible for possible glutathionylation of the subunits [14], which might contribute to rearrangements in the subunits and hence changes in the activity of NKA (Figure 2). 

S-glutathionylation is not limited to α1 subunit of NKA. A cysteine residue close to the transmembrane part of β1-NKA and FXDY is also glutathionylated in oxidative stress, leading to impaired pump activity [111]. Figtree et al. reported that in rabbit ventricular myocytes at baseline levels β1-NKA, but not α1-NKA, is glutathionylated. They also showed that angiotensin II via AT1 receptor stimulation increased β1 subunit glutathionylation, which decreased α1/β1 subunit coimmunoprecipitation and decreased pump activity due to increased oxidation by NAPDH secondary to elevated protein kinase C activity. Similarly, in a sheep model of myocardial infarct regions β1-NKA glutathionylation increased [106,112].

Glutathionylation of one of the subunits can also modify interactions with other subunits. For example, FXDY1 proteins have roles in deglutathionylation of β1-NKA and restore the activity of NKA [111]. These observations indicate that glutathionylation is not limited particularly to subunits or redox regulating agents.

It has been suggested that CTS-mediated redox sensitive NKA activity may be desensitized in the long term and may reduce ion transport capability [113]. However, antioxidant supplementation did not show any benefit, suggesting that redox status of the cells is not the only factor affecting NKA activity in hypoxic cells. Thus, understanding this regulation in the ischemic heart is particularly important for the effects and clinical use of cardiac glycosides.

The changes in posttranslational modification of proteins in cellular signaling have received attention for identifying their roles in pathophysiological conditions. A recent study showed that in porcine renal proximal tubular LLC-PK1 cells, short-term ouabain treatment caused reversible carbonylation of α1-NKA by c-Src-dependent mechanisms and inhibited transepithelial ^22^Na^+^ flux [114]. It is yet unclear whether other posttranslational modifications such as palmitoylation, acetylation of subunits, occur during ischemia and interfere with the interacting protein partners, thus affecting cellular trafficking and stability of the pump [115].

### 8.2. Inflammation-Dependent Regulation of NKA

Inflammation has a central role in the development, progress, and complications of cardiovascular disease such as myocardial infarction (MI) and atherosclerosis. In cases of acute myocardial infarction, immune cells recruit to locally injured areas and disseminate to blood circulation to limit injury and trigger tissue repair and regeneration (recently reviewed [116,117]). Continued inflammation and accompanying tissue hypoxia are associated with worse outcomes. Tissue injury upregulates the release of pro-inflammatory cytokines, TNF-α, IL-6 and IL-1β, lead to activation of platelets that mediate leukocyte activation, recruit neutrophils to myocardial infarct area, damage endothelial cell integrity and function, and cause systemic inflammation [118,119]. Induced cytokine production stimulates the expression of reactive oxygen species (ROS), inducible nitric oxide synthase (iNOS) and nuclear factor kappa B (NFκB), and further modulates cytokine production [116]. ROS is released from the ischemic cardiomyocytes after MI and interacts and changes the function of a variety of enzymes and proteins involved in cellular function.

Inflammation-dependent regulation of NKA and the molecular mechanisms behind it have been reported in the lung, the kidney, and the heart. In a lipopolysaccharide (LPS)-mediated sepsis model, NKA activity decreased in the myocardium in vivo and in vitro in primary neonatal cardiomyocytes. Decreased cardiac function was inhibited by the TNF-α antagonist etanercept. This study further identified that the PI3K/Rac1/NADPH oxidase cascade was involved in inhibiting NKA. Inhibition of NKA-activated Ca^2+/^CaMK/mTOR signaling, promoted myocardial TNF-α elevation and cardiac dysfunction. Interestingly, ouabain treatment of rats aggravated TNF-α production and further suppressed cardiac function in LPS treated mice [120]. ROS also contributed to myocardial TNF-α expression and cardiac dysfunction [121,122]. Furthermore, activation of NADPH oxidase inhibits NKA by glutathionylation of its β-subunit [106] (Figure 2).

Nitric oxide (NO) and nitrite-related products are common intermediate reactive molecules produced by a variety of inflammatory signals. NO and superoxide-dependent peroxynitrite play important roles in mediating NO-related tissue damage and ion transport [123]. Direct application of NO donors nitrosylated the NKA in H441 airway epithelial cells and decreased NKA activity by S-nitrosylation of the protein. Preventing the reaction of NO with thiol groups blunted NKA inhibition [124]. However, as mentioned above acute hypoxia decreases NO production and S-nitrosylation of the NKA [125], shifting the posttranslational modification of subunits towards S- glutathionylation. These findings underline that NKA regulation is more complex in the presence of inflammation associated with hypoxia.

## 9. Cardiotonic Steroids (CTS): More Than Inhibitors of NKA Activity

Cardiotonic steroids (CTS) are used for the treatment of arrhythmias and heart failure in patients with reduced ejection fraction due to their positive inotropic effects. According to a clinical trial “The Digitalis Investigating Group”, CTSs are helpful for relief of symptoms in patients with impaired left ventricular systolic function, without any impact on long-term mortality [126]. Concerns of arrhythmogenic side effects and toxicity due to its narrow therapeutic index, drug interactions such as verapamil, amiodarone, spironolactone and risks of comorbidities such as kidney dysfunction led to a decrease in its utility [127].

Recent studies demonstrated that NKA is not only an ion transporter, but independent of this function it is now also considered a signaling molecule [5,6]. This argument is based on the findings that NKA interacts with caveolae [128], epidermal growth factor receptor (EGFR) [129], ankyrin B [130], Akt substrate of 160 kDa (AS160) [131], Ras superfamily of small GTPases (Ral-GTPase) [132], and G protein-coupled receptor 35 (GPR35) [133] in different tissues and contributes to several signaling events. For instance, in vascular smooth muscle cells, inhibition of NKA by ouabain increased intracellular Ca^+2^ levels via the interaction of NKA with c-Src in a manner independent of intracellular ion concentrations [6]. Disrupting the NKA-related signaling and c-Src activation by a peptide (NaKtide) attenuated cardiac fibrosis [134,135]. In neonatal cardiac myocytes ouabain activated EGFR, PLC, PI3K, and PKC and increased ROS independently of cytosolic Ca^2+^ levels [136]. Additionally, stimulated interaction between NKA and c-Src by ouabain induced the endocytosis of NKA from the plasma membrane [137,138]. It is not yet known how hypoxia affects these signaling pathways. In hypoxic murine fibroblasts ouabain decreased ROS accumulation, did not activate c-Src, and increased cell survival while NKA activity decreased. This has been attributed to changes in the NKA and ouabain interaction [139]. It is unclear whether similar changes also occur in the ischemic myocardium.

In vitro studies showed that inhibition of NKA by CTS increased the transcription of genes related to cardiac hypertrophy [7,140], triggered cardiac fibrosis and decompensation [141,142,143,144]. Skoumal et.al., reported increased levels of an endogenous, ouabain-like compound in the blood from animals treated with norepinephrine and angiotensin II which developed cardiac hypertrophy [145]. After a long debate it is now accepted that endogenous CTS [146,147] is markedly increased in chronic renal failure, hyperaldosteronism, hypertension, congestive heart failure and salt sensitivity [148]. Moreover, during heart failure, the heart becomes more sensitive to the effects of ouabain due to a decreased number of the pump sites [42]. In a model of hypertrophic cardiomyopathy, ouabain worsened diastolic sarcomere length due to high cytosolic Ca^2+^ levels, associated with increased myofilament Ca^2+^ sensitivity and decreased NKA expression [149]. It would be interesting to know whether ouabain inhibits HIFs in the hypoxic heart, as latest research reported their use for the treatment of several cancers where HIFs are involved in tumor growth [150,151,152,153] and also in the treatment of pulmonary hypertension [154] (Figure 2 and Figure 3).

Based on these studies one might put forward the hypothesis that use of CTS in heart failure in the presence of increased plasma levels may further aggravate the disease progression through a variety of signaling cascades. Depending on the recent findings with sodium-glucose cotransporter 2 (SGLT2) inhibitors’ effect on modulating NHE1 and NCX activities, improving Na^+^ and Ca^2+^ handling, and preventing cardiac remodeling while maintaining ATP content [16,113,155], decreasing intracellular Na^+^ and Ca^2+^ overload in heart failure might be considered an alternative approach.

## 10. Conclusions and Perspectives

Our current understanding is that NKA activity is impaired in ischemic heart disease. While it appears clear that the decrease in NKA activity depends on hypoxia, the underlying mechanisms behind this and the regulation of subunit changes are still uncertain. It also remains to be identified whether the hypoxic response is mediated by HIF prolyl-hydroxylase-dependent signaling in regulating the NKA and whether the inconsistent results on the expression and activity of NKA depend on different degrees of hypoxic stress induced by the experimental models.

Decreased NKA activity in the ischemic heart contributes to intracellular Na^+^ and Ca^2+^ dysregulation and causes diastolic dysfunction. Therefore, increasing the NKA activity might alleviate cardiac stress. Considering the beneficial effects of SGLT2 inhibitors on improving cardiac function, their effect on NKA should also be investigated on non-diabetic ischemic heart models.

The limited benefits of the currently used treatment strategies in the treatment of ischemic heart disease point to reconsidering different impaired signaling pathways for the development of new therapeutic strategies along with exercise interventions and life-style changes. Furthermore, the reduced activity of NKA in heart failure might be aggravated by CTS treatment due to increased intracellular Ca^2+^ levels, secondary to increased Na^+^ that may potentially lead to damage of myocytes. Based on this, the effects of endogenous, or exogenous CTS in the ischemic heart is required to be identified for their precise use in the clinics (Figure 3).

## Data Availability

Not applicable.
